# Using Smartphones to Monitor Bipolar Disorder Symptoms: A Pilot Study

**DOI:** 10.2196/mental.4560

**Published:** 2016-01-06

**Authors:** Till Beiwinkel, Sally Kindermann, Andreas Maier, Christopher Kerl, Jörn Moock, Guido Barbian, Wulf Rössler

**Affiliations:** ^1^ Innovation Incubator Competence Tandem Integrated Care Leuphana University of Lüneburg Lüneburg Germany; ^2^ Center for Psychotherapy Research University Hospital Heidelberg Heidelberg Germany; ^3^ Institute of Knowledge and Information Management Leuphana University of Lüneburg Lüneburg Germany; ^4^ Institute of Psychiatry Laboratory of Neuroscience (LIM 27) University of Sao Paulo Sao Paulo Brazil

**Keywords:** smartphone, sensor technology, bipolar disorder, monitoring, phase transitions, communication patterns, activity patterns

## Abstract

**Background:**

Relapse prevention in bipolar disorder can be improved by monitoring symptoms in patients' daily life. Smartphone apps are easy-to-use, low-cost tools that can be used to assess this information. To date, few studies have examined the usefulness of smartphone data for monitoring symptoms in bipolar disorder.

**Objective:**

We present results from a pilot test of a smartphone-based monitoring system, Social Information Monitoring for Patients with Bipolar Affective Disorder (SIMBA), that tracked daily mood, physical activity, and social communication in 13 patients. The objective of this study was to investigate whether smartphone measurements predicted clinical symptoms levels and clinical symptom change. The hypotheses that smartphone measurements are (1) negatively related to clinical depressive symptoms and (2) positively related to clinical manic symptoms were tested.

**Methods:**

Clinical rating scales were administered to assess clinical depressive and manic symptoms. Patients used a smartphone with the monitoring app for up to 12 months. Random-coefficient multilevel models were computed to analyze the relationship between smartphone data and externally rated manic and depressive symptoms. Overall clinical symptom levels and clinical symptom changes were predicted by separating between-patient and within-patient effects. Using established clinical thresholds from the literature, marginal effect plots displayed clinical relevance of smartphone data.

**Results:**

Overall symptom levels and change in clinical symptoms were related to smartphone measures. Higher overall levels of clinical depressive symptoms were predicted by lower self-reported mood measured by the smartphone (beta=-.56, *P*<.001). An increase in clinical depressive symptoms was predicted by a decline in social communication (ie, outgoing text messages: beta=-.28, *P*<.001) and a decline in physical activity as measured by the smartphone (ie, cell tower movements: beta=-.11, *P*=.03). Higher overall levels of clinical manic symptoms were predicted by lower physical activity on the smartphone (ie, distance travelled: beta=-.37, *P*<.001), and higher social communication (beta=.48, *P*=.03). An increase in clinical manic symptoms was predicted by a decrease in physical activity on the smartphone (beta=-.17, *P*<.001).

**Conclusions:**

Clinical symptoms were related to some objective and subjective smartphone measurements, but not all smartphone measures predicted the occurrence of bipolar symptoms above clinical thresholds. Thus, smartphones have the potential to monitor bipolar disorder symptoms in patients’ daily life. Further validation of monitoring tools in a larger sample is needed. Conclusions are limited by the low prevalence of manic and depressive symptoms in the study sample.

**Trial Registration:**

International Standard Randomized Controlled Trial Number (ISRCTN): 05663421; http://www.controlled-trials.com/ISRCTN05663421 (Archived by WebCite at http://www.webcitation.org/6d9wsibJB)

##  Introduction

Bipolar disorder is a serious and disabling psychiatric condition that encompasses a broad group of disorders. International lifetime prevalence estimates indicate that bipolar disorders are present in 1-5% of the general population [1]. It involves mood, behavioral, and cognitive disruptions during episodes of depression, mania, or hypomania. The recurrent and chronic nature of bipolar disorder results in a high burden of disease [2-4] and high societal costs [5]. The suicide rate of people diagnosed with bipolar disorder is the highest among all mental disorders [4,6]. Even during periods of remission, patients experience frequent subclinical mood symptoms that impair daily functioning and increase their risk for relapse [7,8].

The heterogeneity of symptoms and individual courses of disease in bipolar disorder make it difficult to predict the course of the disorder [9]. Compared to a patient with high blood pressure who needs to maintain blood pressure below a certain threshold, no analogous guidance is currently provided for patients suffering from bipolar disorder. As a consequence, patients often do not recognize their mood changes in a timely manner and lose their insight into illness [10], leading to adverse consequences [11]. In order to prevent relapses, timely information on upcoming phase transitions must be available to patients and doctors [12]. This information could allow providers to intervene shortly after symptoms appear.

Information from patients’ daily life can help provide an earlier and more reliable prediction of impending phase transitions in bipolar disorder [13-16]. It has been suggested that smartphones may be easy-to-use, low-cost devices that can be used to measure this information in the patient’s daily environment. Using both self-reported information collected by the device and making use of the smartphone sensor capabilities, researchers hope to gain insight in the user’s well-being and behavior. Among mental health patients, too, there is great interest in monitoring symptoms with mobile apps [17]. It has been found that daily mood and the level of physical and social activity can be measured with smartphone sensors [18-22]. These measurements are assumed to represent central aspects of bipolar disorders [23,24].

Additional research is needed to establish the relationship between smartphone measurements and clinical symptoms in bipolar disorder. In particular, a more personalized approach to capture warning signs for impending phase transitions needs to consider both the patients’ overall symptom levels and the dynamic symptom changes occurring over time. The advantage of this approach is that it is able to capture the interindividual variability and heterogeneity of bipolar disorder, where symptom severity within one patient fluctuates over time [25]. While statistical methods exist to compute predictions for overall symptom levels and symptom change in smartphone data [26], previous studies did not separate between these central illness components.

To address this research gap, a study was conducted to investigate whether smartphone data predict impending clinical symptoms in bipolar disorder. The aim of this study is twofold. First, we compare the relationship of daily self-reported mood, smartphone sensor data (ie, Global Positioning System [GPS], cell tower movements, accelerometer), and smartphone communication (ie, calls, short message service [SMS] log) with clinical symptoms. We hypothesize that for depressive symptoms, higher levels of self-reported mood, and higher levels of physical activity and social communication measured by the smartphone predict lower overall levels of depressive symptoms and temporal decreases in clinical depressive symptoms. For manic symptoms, we hypothesize that higher levels of self-reported mood and higher levels of physical activity and social communication measured by the smartphone predict both higher overall levels of clinical manic symptoms and temporal increases in manic symptoms. Second, to identify the clinical relevance of smartphone data we test the hypothesis that smartphone data predict the occurrence of overall symptoms levels above clinical thresholds.

##  Methods

### Recruitment

**Figure 1:**
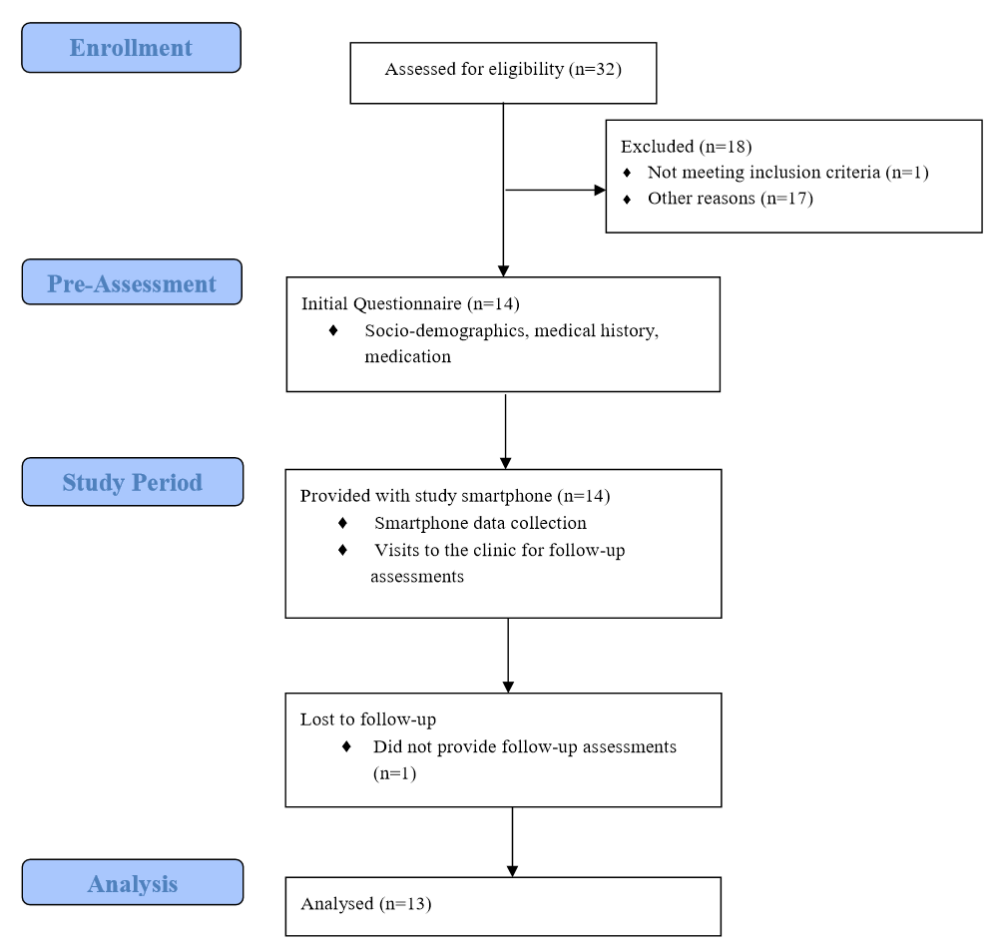
Study flow chart.

Figure 1 presents the study flow chart. Participants were recruited from the outpatient department of a Psychiatric Clinic in Lower Saxony, Germany, between July 2013 and February 2014. Patients were contacted and assessed for eligibility in random order from the pool of outpatients. Inclusion criteria were diagnosis of bipolar I or bipolar II disorder according to the criteria in the *Diagnostic and Statistical Manual of Mental Disorders*, 4^th^ edition (DSM-IV) [27], at least 18 years of age, sufficient knowledge of the German language, and basic competence in using mobile devices. Exclusion criteria were the need for inpatient treatment at the time of recruitment, suicidality, diagnosis of schizophrenia or an intellectual disability, and alcohol or drug abuse up to 6 month prior to the study. All participants provided written consent prior to participation and were loaned a smartphone for the 12-month study period. Participants were provided with an unlimited call, text, and data plan and were encouraged to use the study smartphone as their regular mobile device. The study was examined and approved by the Leuphana University Ethics Committee.

### Measurements

Smartphone data were collected on a Sony Ericsson Xperia Neo V smartphone with Android 4.0.4. The device had the Social Information Monitoring for Patients with Bipolar Affective Disorder (SIMBA) app pre-installed. The app was developed by the authors and is based on two open source frameworks for the collection of both subjective self-report data [28] and objective sensor data [29], which are available at [30,31]. First, Open Data Kit developed at the University of Washington [28] collects subjective self-report data. Designed for socioeconomic and health surveys, Open Data Kit provides a platform to build questionnaires and to collect data on a smartphone. Its modular design allows for the implementation of questionnaires at fixed or random time points. Second, the Funf open sensing framework developed at the Massachusetts Institute of Technology (MIT) media lab [29] collects objective data using various smartphone sensors, for example, GPS, accelerometer, and screen state. It provides an extensible sensing and data processing framework for smartphones. As using smartphone sensors can drain the device battery quickly, Funf is designed to prolong battery life. The source code for the software frameworks can be found in Multimedia Appendix 1.

Three smartphone sensors were used to measure physical activity: GPS for the distance traveled per day, cell tower movement as an indicator of location changes, and accelerometer to measure the users’ device activity. Both GPS and cell tower movements capture spatial movement and since the correlation between GPS and cell tower movements was low (*P*=.06), both measures were included. For the measurement of social communication, the number and duration of outgoing calls and the number of SMS sent per day were logged. The gathered data were cached in local storage and transferred to a secured server located at Leuphana University whenever an Internet connection was available.

Self-reported mood states were assessed once a day on the smartphone at random times with a 2-item questionnaire. When the questionnaire was available, participants were notified via a beep and asked if the questionnaire should be answered now or if the participant wished to be notified later. Affect was assessed with the item, “On a scale of 1 (very good) to 10 (very bad), please describe your present mood,” and energy was assessed with the item, “On a scale of 1 (very energetic) to 10 (not at all), how energetic do you feel at the moment?” For the purposes of this analysis, both items were reverse-coded so that higher scores reflected better mood. Due to the high correlation between the 2 items (*P*=.84), a single mood index was constructed.

Clinical assessments were conducted repeatedly throughout the study and served as a reference point for the smartphone data. Appointments with the treating clinician, who was blinded to smartphone data, were scheduled at approximately 8-week intervals and included assessment of manic and depressive symptoms using clinical rating scales. Manic symptoms were assessed using the German Version [32] of the Young Mania Rating Scale (YMRS) [33]. The scale is administered by a clinician and rates major relevant manic symptoms (eg, elevated mood, motoric activity) on a scale from 0-64. Values less than 5 indicate complete remission [34]. Depressive symptoms were assessed with the German Version [35] of the Hamilton Depression Scale (HAMD) [36]. The scale is based on clinician assessment and scores 17 items within a reference period of 1 week. Values greater than 7 indicate at least a mild depressive syndrome [37].

### Statistical Analysis

Smartphone data were aggregated to assessment periods before each clinical appointment by computing mean scores from the daily measurements resulting in an aggregated score for each assessment period. An assessment period is the number of days between two clinical appointments. Then, the effect of the aggregated smartphone data on the clinical symptoms was assessed for each assessment period. To accommodate the nested data structure, where observations are nested within patients, multilevel models were computed according to [38] in order to obtain robust and unbiased estimates of variance components. Random coefficient models were fitted, where assessment periods were nested within patients. Smartphone data were modeled to separate between-patient effects (ie, symptom level) and within-patient effects (ie, symptom change) [26].

We estimated three separate models for self-reported data, activity data, and social data, and one full model including all smartphone data. All models were adjusted for age, sex, and length of assessment period. The total length of assessment periods was used in order to compute more precise estimates, as opposed to restricting the analysis to the shorter time frame using the reference period of HAMD and YMRS before each clinical assessment. To compare the results of the models, standardized regression coefficients are reported [39]. To provide a graphical representation of the relationship between smartphone data and clinical symptoms, the results of the full model were used to compute marginal effects, which were plotted against symptoms. The analysis was performed using the “xtmixed” command in Stata 13, where the maximum likelihood estimation method was specified [40]. Significance levels are reported at the 95% level.

## Results

### Sample Characteristics

Of the 14 patients who were recruited, one dropped out before clinical follow-up assessment and was thus removed from the study. Table 1 presents the sample characteristics for the 13 patients who completed the study.

### Description of Dataset

Table 2 shows the available data points for self-reported data, activity data, social data, and clinical data. The compliance rate for self-reported data was 55.7%. Activity data were complete on 78.2% of days, and social data were present on 56.1% of days.

### Mean Levels of Smartphone and Clinical Data

Table 3 presents mean levels and range for smartphone-collected indicators and clinical data. The average level of self-reported mood was 6.7 (SD 1.7) with a maximum of 10, indicating that average mood levels of subjects were at the upper end of the end of the scale ranging from 1 (very bad mood) to 10 (very good mood). Clinical symptom ratings revealed mean manic symptoms levels assessed by YMRS of 2.7 (SD 3.6) and depressive symptoms assessed by HAMD of 5.1 (SD 5.3), indicating low prevalence of manic and depressive symptoms in the sample.

**Table 1:** Sample characteristics for 13 patients.

Characteristic		N	Value
Age in years, mean (SD)		13	47.2 (3.8)
**Sex, %**			
	Male	8	61.5
	Female	5	38.5
**Education, %**			
	Lower secondary	9	69.2
	Upper secondary	4	30.8
**Qualification**			
	None	1	7.7
	Vocational	10	76.9
	University level	2	15.4
**Employed, %**			
	Yes	4	30.8
	No	9	69.2
**Diagnosis, %**			
	Bipolar I	6	46.1
	Bipolar II	7	53.9
Years since first diagnosis, mean (SD)		13	9.9 (3.1)
Manic episodes, mean (SD)		13	8.6 (1.6)
Depressive episodes, mean (SD)		13	12.3 (3.0)
Total hospitalizations, mean (SD)		13	6.9 (2.8)
**Currently medicated, %**			
	Yes	11	84.2
	No	2	15.4
Study duration in days, mean (SD)		13	365.1 (31.9)
Length of assessment period, mean (SD)		13	68.6 (23.6)

**Table 2:** Total available data points for self-reported data, activity data, social data, and clinical data collected by 13 patients.

	N	Mean (SD)	Min/Max	Rate, %
Self-reported	2456	188.9 (83.3)	24/291	55.7
Activity	3537	272.1 (74.9)	154/362	78.2
Social	2624	201.8 (109.3)	9/339	56.1
Clinical	75	5.8 (1.4)	3/8	NA

**Table 3:** Mean levels, interquartile range, and range for smartphone indicators and clinical variables for 13 patients.

Variable		Mean (SD)	Interquartile Range	Min/Max
**Self-reported**				
	Mood	6.7 (1.7)	2.0	1/10
**Activity**				
	Distance traveled, km	10.5 (41.5)	6.3	0/732
	Cell tower changes	10.5 (17.0)	10.0	0/139
	Device activity, % of day	7.3 (8.2)	9.2	0/75
**Social**				
	Number outgoing calls	2.9 (3.6)	4.0	0 /29
	Duration outgoing calls, minutes	10.2 (19.6)	11.3	0/181
	Outgoing SMS	1.7 (3.6)	2.0	0/54
**Clinical**				
	YMRS	2.7 (3.6)	4.0	0/18
	HAMD	5.1 (5.3)	9.0	0/18

### Prediction of Clinical Symptoms

This section presents the results from the multivariate models with smartphone data as predictors for clinical symptoms. We begin with results from the between-patients analysis, representing the overall level of clinical symptoms as predicted by smartphone data. Next, we present results of the within-patient analysis, representing temporal changes in clinical symptoms as predicted by smartphone data.

#### Between-Patient Analysis

The combined model (Model 4) in Table 4 showed a significant negative relationship between mood level and clinical depressive symptoms (HAMD: beta=-.56, *P*<.001) but not on manic symptoms. This suggests that patients who reported better daily mood on the smartphone were less clinically depressed. In the activity data, distance traveled as measured by the GPS signal had a significant negative relationship with clinical manic symptoms (YMRS: beta=-.37, *P*<.001), indicating that patients who were more physically active experienced fewer manic symptoms. Cell tower movements and device activity were not significantly related to any clinical symptoms. In the social data, the number of calls made on the smartphone were positively related to clinical manic symptoms (YMRS: beta=.48, *P*=.03), suggesting that patients who made a higher frequency of calls experienced higher levels of manic symptoms. The duration of calls was not significantly related to symptoms. The number of outgoing SMS had a negative relationship with clinical depressive symptoms (HAMD: beta=-.17, *P*<.001), indicating that patients who sent more SMS had less severe depressive symptoms.

**Table 4:** Between-patient relationship of self-reported data, activity data, and social smartphone data with bipolar disorder symptoms for 13 patients based on 75 clinical ratings^a^.

		Beta (*P*)			
		Self-report	Activity	Social	Combined
		Model 1 (N=74)	Model 2 (N=62)	Model 3 (N=71)	Model 4 (N=62)
**Mood**					
	YMRS	-.09 (.45)			.05 (.79)
	HAMD	-.42 (<.001)			-.56 (<.001)
**Distance traveled, km**					
	YMRS		-.46 (.01)		-.37 (<.001)
	HAMD		-.24 (.12)		-.12 (.34)
**Cell tower movements**					
	YMRS		-.24 (.29)		-.14 (.12)
	HAMD		.08 (.58)		-.04 (.82)
**Device activity, %**					
	YMRS		.31 (.24)		.26 (.12)
	HAMD		-.01 (.92)		-.06 (.80)
**Number of calls**					
	YMRS			.19 (.38)	.48 (.03)
	HAMD			.34 (.17)	.08 (.65)
**Duration of calls, minutes**					
	YMRS			.17 (.45)	-.08 (.72)
	HAMD			-.22 (.25)	.03 (.83)
**Outgoing SMS**					
	YMRS			.03 (.72)	-.02 (.65)
	HAMD			.04 (.84)	-.17 (<.001)

^a^Standardized effects of random coefficient regression models with smartphone data as predictor of depressive symptom levels (YMRS) and manic symptom levels (HAMD).

#### Within-Patient Analysis

Table 5 displays the within-patient relationship between change in smartphone data and change in clinical symptoms. In the full model, change in self-reported mood on the smartphone was not related to clinical symptom changes. An increase in cell tower movement was negatively related to both manic symptoms (YMRS: beta=-.17, *P*<.001) and depressive symptoms (HAMD: beta=-.11, *P*=.03), suggesting that when a patient’s activity level as measured by the smartphone increased, both manic and depressive symptoms decreased. However, changes in distance traveled and device activity were not significantly related to symptom changes. In the social data, an increase in outgoing SMS was negatively related to a change in depressive symptoms (HAMD: beta=-.28, *P*<.001), which suggests that when more SMS are sent, clinical depressive symptoms are lowered.

**Table 5:** Within-patient relationship of change in self-report, activity, and social smartphone data with change in bipolar disorder symptoms for 13 patients^a^.

		Beta (*P*)			
		Self-report	Activity	Social	Combined
		Model 1 (N=74)	Model 2 (N=62)	Model 3 (N=71)	Model 4 (N=62)
**Mood**					
	YMRS	-.09 (.28)			.03 (.73)
	HAMD	-.18 (.10)			-.09 (.26)
**Distance traveled, km**					
	YMRS		.03 (.40)		.01 (.85)
	HAMD		.07 (.23)		.03 (.66)
**Cell tower movements**					
	YMRS		-.10 (.03)		-.17 (<.001)
	HAMD		-.17 (<.001)		-.11 (.03)
**Device activity, %**					
	YMRS		-.11 (.17)		-.07 (.26)
	HAMD		-.15 (.09)		.02 (.87)
**Number outgoing calls**					
	YMRS			.18 (.34)	.24 (.44)
	HAMD			-.07 (.60)	-.07 (.73)
**Duration outgoing calls, minutes**					
	YMRS			-.25 (.27)	-.34 (.24)
	HAMD			-.07 (.63)	-.09 (.58)
**Outgoing SMS**					
	YMRS			-.05 (.35)	.03 (.68)
	HAMD			-.30 (<.001)	-.28 (<.001)

^a^Standardized effects of random coefficient regression models with smartphone data as predictor of depressive symptom change (YMRS) and manic symptom change (HAMD).

### Clinical Relevance of Smartphone Data

**Figure 2:**
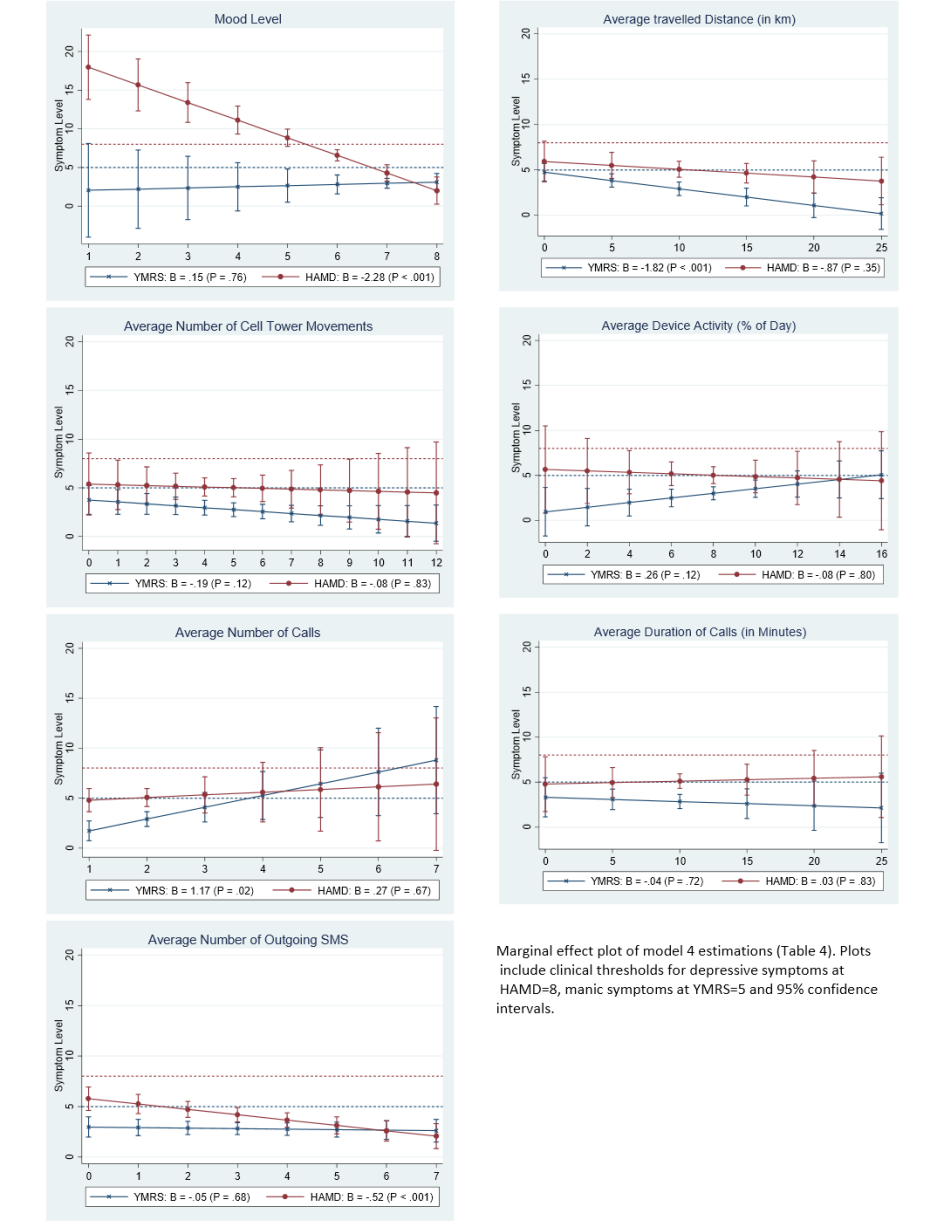
Between-patient analysis of smartphone data and symptom levels.

**Figure 3:**
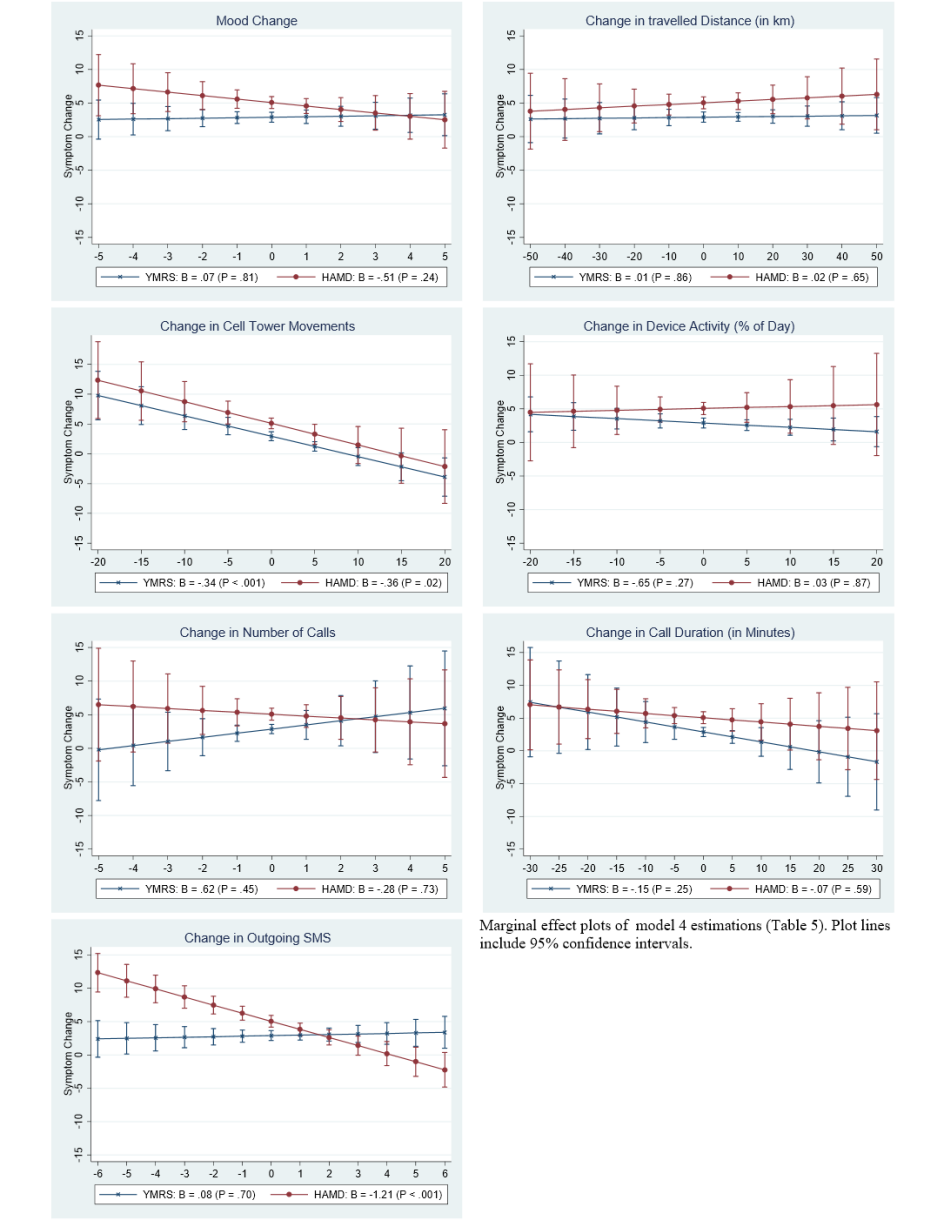
Within-patient analysis of smartphone data and symptom change.

Figure 2 plots predicted symptoms levels including clinical thresholds, and Figure 3 plots predicted symptom change by smartphone data. The plots are computed from the full models in Tables 4 and 5.

#### Between-Patient Analysis

Figure 2 shows that depressive symptoms above the clinical threshold are predicted when the average mood level is lower than 6 points on the 10-point mood scale. While the average number of calls had a positive effect on clinical manic symptoms (YMRS: beta=1.17, *P*=.02) and the average number of outgoing SMS had a negative relationship with clinical depressive symptoms (HAMD: beta=-.52, *P*<.001), this did not predict the occurrence of symptoms above the clinical threshold.

#### Within-Patient Analysis

Figure 3 visualizes the relationship between change in smartphone data and change in clinical symptoms as computed in Table 5. The negative relationship of change in cell tower movements with both clinical manic symptoms (YMRS: beta=-.34, *P*<.001) and clinical depressive symptom changes (HAMD: beta=-.36, *P*<.02), indicated that when a patient’s activity as measured by the smartphone was below average, clinical symptoms were elevated and vice versa. Figure 3 shows that when a patient made 20 cell tower movements fewer than average, the model predicts an YMRS score of approximately 10 points and an HAMD score of 12 points above the patient’s average. Last, Figure 3 visualizes the negative relationship between change in outgoing SMS and change in depressive symptoms (HAMD: beta=-1.21, *P*<.001), implying that when a patient sent more text messages than average, symptoms were lowered.

## Discussion

### Principal Results

In this pilot study, smartphone-based monitoring of mood, physical activity, and social communication was conducted in the daily life of bipolar disorder patients over the course of 12 months. The between-patient and within-patient variance of smartphone data was analyzed to present the relationship of smartphone data with both overall levels and changes in clinical symptoms. The results allow conclusions on the usefulness of smartphone measurements for the monitoring of bipolar disorder.

Higher overall levels of depressive symptoms were predicted by lower self-reported mood measured by the smartphone. An increase in depressive symptoms was predicted by a decline in social communication (ie, outgoing SMS) and a decline in physical activity (ie, cell tower movements). In contrast to our hypothesis, self-reported mood did not predict clinical manic symptoms. The overall level of manic symptoms was predicted by activity (ie, distance traveled) and social communication (ie, number of calls). In contrast to our hypothesis, an increase in clinical manic symptoms was predicted by lower physical activity (ie, cell tower movements). Other smartphone measurements (ie, device activity and the duration of calls) were related to neither symptom levels nor symptom changes.

In addition, the clinical relevance of the results was examined by investigating if smartphone measurements predicted symptom levels above clinical thresholds. This information may provide information on which smartphone measurements can be used to predict the occurrence of symptoms above clinical thresholds. Self-reported mood was found to predict depressive symptom levels above the clinical threshold but not manic symptoms. No other smartphone measurements predicted symptoms above clinical thresholds.

### Comparison With Prior Work

Within the self-reported data, our data support the findings [14,20] that daily mood ratings are useful for monitoring depressive symptoms in patients who experience bipolar disorder. Similar to those studies, we could not support the hypothesis that daily mood measured by the smartphone predicts clinical manic symptoms. This is probably the result of the low prevalence of patients with severe manic symptoms in our sample (see Table 3).

Physical activity was included in the analysis as it represents a warning sign for phase transitions in bipolar disorder and can potentially be measured objectively with smartphone sensors. Similar to the approach in Faurholt-Jepsen et al [20], we used GPS sensors to track the distance traveled, patient movements across cell towers as a second measurement of physical activity, and the smartphone accelerometers to assess patients’ interaction with the device. In our analysis, some within-patient and between-patient effects showed significant relationships with clinical symptoms. However, we did not expect to find that physical activity was negatively related to manic symptoms, indicating that patients with higher overall levels of physical activity (ie, as measured by distance traveled) experienced fewer manic symptoms. We also did not expect to find that declining temporal activity (ie, as measured by cell tower movements) was associated with reduced manic symptoms. These findings run counter to the literature on the early warning signs of bipolar disorder [41,42], which assumes that increased activity is a prodrome of mania. These diverging results might be explained by the low prevalence of manic symptoms in our study sample. It can be speculated that for patients with subclinical manic symptoms, as in this study, increased activity signals better patient condition, but should not be mistaken with hyperactivity in patients with severe mania.

The social data measured by the smartphones capture patients’ level of interaction with their social surroundings as an early-warning sign of bipolar disorder symptoms. Both between- and within-patient effects of social communication on clinical symptoms were found in the data, implying that overall levels and dynamic changes in communication are relevant for the prediction of bipolar disorder symptoms. The overall level of text messages sent and the number of calls placed indicated levels of depression and manic symptoms, respectively, while changes in text messages sent predicted changes in depressive symptoms. As a larger number of calls, but not the duration of calls, was associated with manic symptoms it is possible that patients with increased manic symptoms have a larger activity mirrored by more calls but not the ability to concentrate on lengthier calls. Overall, our research is in line with findings from other research that highlights the role of psychosocial variables in the illness course in bipolar disorder [43]. However, a comparable study [20] did not find significant correlations between social data and clinical symptoms.

A major feature of this analysis was that the collection of smartphone data and repeated clinical measures allowed us to separate two essential components of illness activity: overall symptom levels as observed by comparing the variance between patients, and dynamic changes that occur over the course of the illness as observed by comparing the variance within one patient over time. Although this approach introduces additional assumptions to the model and can make the interpretation of results more challenging, theoretical reasons speak for the separation of between- and within-patient variance. Evidence from modern follow-up studies shows that the course of illness in bipolar disorder is characterized by high interindividual variability and heterogeneity [25]. Symptom severity within one patient fluctuates over time and often includes the expression of major, minor, and subclinical symptoms at different stages of illness [7]. Between-patient comparisons are insufficient to analyze how symptoms develop over time as they do not capture these within-person processes. The repeat sampling rate of the smartphone data in this study offers the advantage that the dynamic nature of psychopathology can be studied in real-time and in the real world.

### Limitations

This study had several limitations. The small sample size of 13 patients may have lowered the statistical power of the study, leading to type II errors in the statistical conclusions. The number of participants did not allow the inclusion of additional level 2 predictors, which might explain between-patient differences (eg, type of bipolar diagnosis). Therefore, it is necessary to replicate the study in a larger sample to validate use of smartphone data for clinical applications.

The low prevalence of clinical depressive and manic symptoms (see Table 3) in our sample may have prevented us from detecting effects that would have been present in a sample of patients who experienced more severe symptoms. The generalization of the results to patients with more severe symptoms should be made carefully. Patients who are more severely ill may show different levels of acceptance in using smartphones for symptom monitoring. It is possible that the patients recruited for this study were particularly motivated to use smartphones.

Finally, compliance with filling out self-reporting data may have affected the results. However, we did not observe a decline in compliance with self-reported mood over time, indicating that missing values were missing at random. The compliance rate (55.7%; see Table 2) in our study is comparable to a study by Depp et al [44]. The conclusions of the social data could have been limited by the fact that we were unable to assess communication over social media (eg, WhatsApp, Facebook). With communication habits moving towards social media apps, social media should be included in patient monitoring systems. We cannot exclude the possibility that patients shared the device with other people, although patients were instructed accordingly and no indication of such usage was found. Regarding the operating system, monitoring was restricted to Android smartphones and future studies should implement software for other operating systems (eg, iOS, Windows Phone) as well.

### Ethical Considerations

Symptom monitoring in a patient’s daily life involves the collection of sensitive health-related data including communication habits and movement patterns. Sensor-based data, such as GPS locations, need to be protected from unauthorized access. As such, concerns regarding data privacy and confidentiality issues need to be taken seriously in order to guarantee the safety of the collected personal data and to build patient trust. In this study, the encrypted and anonymized transmission of smartphone data to a protected server proved to be successful in ensuring patient data safety. From our experience, the full disclosure of the functionality of the monitoring app, as well as its potential clinical application, was also critical in fulfilling the ethical requirements.

### Conclusions

Symptom monitoring is an important strategy to prevent relapses in patients with bipolar disorder. Smartphone apps are easy-to-use, low-cost tools that assist with symptom monitoring in daily life. In this pilot study, we tracked patient mood, levels of physical activity, and social communication over 12 months with an Android-based monitoring software (SIMBA). To our knowledge, this is the first study that successfully embedded a smartphone-based monitoring strategy in patients’ daily life over such a long time frame. The study provides encouraging results concerning the feasibility, data analytic approaches, and clinical relevance of smartphone-based monitoring for bipolar disorder. With further clinical validation of smartphone data, it may be possible to provide smartphone-based monitoring tools for routine care, which may benefit patients and doctors.

## Appendix

Multimedia Appendix 1 Source Code.

## Abbreviations

DSM-IV: Diagnostic and Statistical Manual of Mental Disorders, 4th edition
HAMD: Hamilton Depression Rating Scale
SIMBA: Social Information Monitoring for Patients with Bipolar Affective Disorder
YMRS: Young Mania Rating Scale
